# Cohort Profile: The UK Women’s Cohort Study (UKWCS)

**DOI:** 10.1093/ije/dyv173

**Published:** 2015-10-01

**Authors:** Janet E Cade, Victoria J Burley, Nisreen A Alwan, Jayne Hutchinson, Neil Hancock, Michelle A Morris, Diane E Threapleton, Darren C Greenwood

**Affiliations:** 1 School of Food Science and Nutrition,; 2 Leeds Institute for Cardiovascular and Metabolic Medicine, University of Leeds, Leeds, UK,; 3 Department of Health Sciences, University of York, York, UK,; 4 School of Public Health and Primary Care, Chinese University of Hong Kong, Hong Kong,; 5 Academic Unit of Primary Care and Population Sciences, Faculty of Medicine, University of Southampton, Southampton, UK and; 6 School of Geography, University of Leeds, Leeds, UK

## Why was the cohort set up?

The UK Women’s Cohort Study (UKWCS) was established to explore links between diet and chronic disease, in particular cancer. Previous cohort studies exploring diet and cancer have often produced results with small, not statistically significant effect sizes, due in part to the fact that diet is a complex exposure with measurement being subject to a varietyof errors and bias.^1–4^ This measurement error has limited our ability to make dietary recommendations linked to chronic disease prevention,^5^ and many important questions remain unanswered. In addition, within population subgroups, diet often appears homogeneous, preventing any subtle effects of dietary differences from being detected. The UKWCS aimed to address these issues in a number of ways. Dietary information was obtained using two methods: a food frequency questionnaire (FFQ) and also a 4-day food diary to provide alternative measures of diet to allow for sensitivity analyses and potential minimization of measurement error. Participants for inclusion in the cohort were selected with a wide range of dietary patterns to maximize dietary variation. The cohort was constructed to have similar, large numbers of subjects in three main groups: vegetarian, eating fish (not meat) and meat-eaters. This design ensured higher power to explore potential relationships between diet (foods, nutrients and dietary patterns) and chronic disease outcomes with appropriate analysis, ensuring results could be applied to the general population.

The original cohort was planned to have two phases: first the baseline data collection (1995 to 1998) using a postal questionnaire including a detailed FFQ, developed from the FFQ used in the Oxford arm of the European Prospective Investigation into Cancer and Nutrition (EPIC) study.^6^ Phase 2 data were collected 4 years later (1999 to 2002), and included a 4-day food diary, 1-day activity diary and lifestyle questionnaire. Details of all participants were submitted to the Office of National Statistics to be flagged on the NHS Central Register (NHSCR) (now the Health and Social Care Information Centre, HSCIC) using their National Health Service (NHS) number, full name and date of birth where possible.

The aims of the UKWCS were to explore relationships between diet (including foods, nutrients, dietary supplements, dietary patterns and diet costs) and chronic disease (including cancer, cardiovascular disease, obesity and other health outcomes).

## Who is in the cohort?

**Figure 1.**
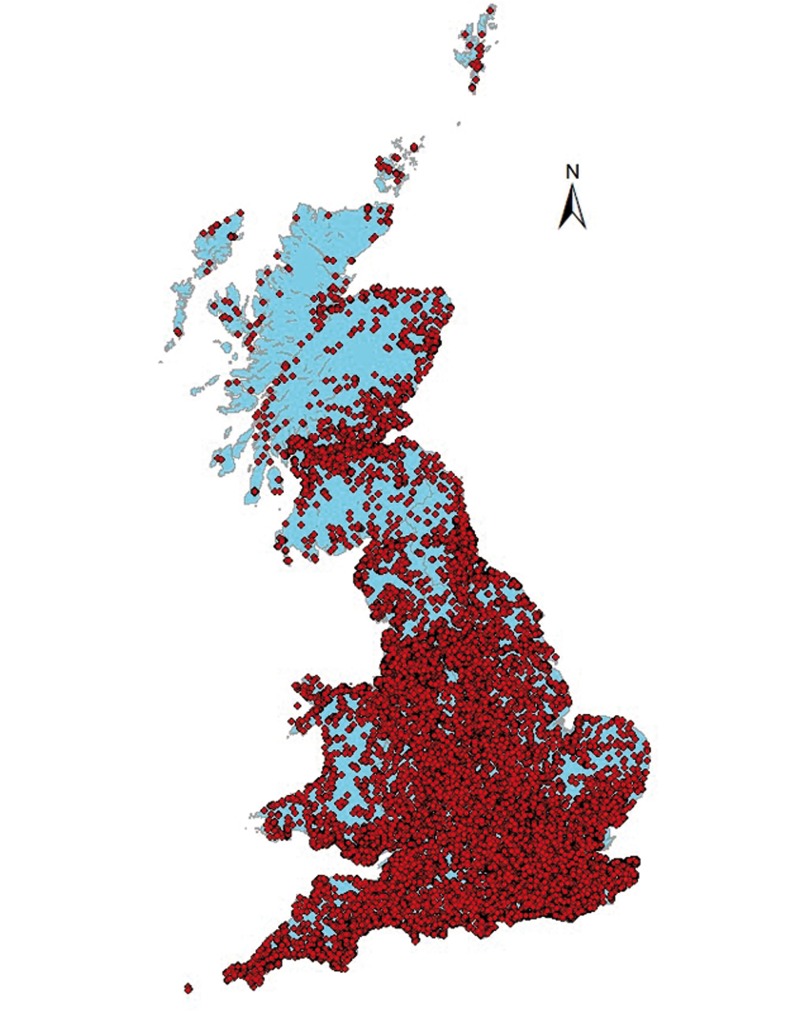
Distribution of the UK Women’s Cohort Study in England, Scotland and Wales.

Women were selected from approximately 500 000 responders to a direct mail questionnaire sent by the World Cancer Research Fund to people living in England, Wales and Scotland, using direct mail lists targeted towards females (Figure 1). These mail lists included subscribers to other, similar charities; 85% of the responders were women and 75% of the responders to the original survey indicated that they would be willing to participate in a more detailed survey. These women formed the population to be contacted to become part of the UK Women’s Cohort. Selection criteria for the cohort included women who self-reported being vegetarian or were non red-meat-eaters and were aged 35–69 years at the time of completion of the direct mail survey. All women in the correct age range and who characterized themselves as vegetarian or non red-meat eaters were eligible to take part. A comparison group was selected by choosing, for each vegetarian, the next non-vegetarian from the stored direct mail database who was aged within 10 years of the vegetarian.

A total of 174 local research ethics committees were contacted and permission to carry out the baseline UKWCS study was obtained.^7^^,^^8^ Participants had consented to the use of information gathered at baseline, future phases and cancer registries for research purposes provided that confidentiality was maintained. In all 61 000 women were mailed the baseline postal questionnaire between 1995 and 1998, and a total of 35 372 women returned this, a response rate of 58%. Women were from across the whole of England, Scotland and Wales (Table 1). The National Research Ethics Committee for Yorkshire and the Humber, Leeds East, gave approval for the recent analysis of cardiovascular disease and have now taken on responsibility for the ongoing cohort.

**Table 1.** Geographical distribution of the UK Women’s Cohort Study compared with the female population of the UK, by Government Office Region

Government Office Region	UK population 2001, females (%)	UKWCS population at baseline, females (%)	UKWCS population as % of UK females
North East	1 296 863 (4)	974 (3)	0.08
North West	3 470 810 (12)	3038 (9)	0.09
Yorkshire and the Humber	2 552 889 (9)	2561 (8)	0.10
East Midlands	2 123 316 (7)	2405 (7)	0.11
West Midlands	2 692 197 (9)	2534 (8)	0.09
East of England	2 749 805 (9)	3001 (9)	0.11
Greater London	3 703 298 (13)	3709 (11)	0.10
South East	4 095 490 (14)	6789 (21)	0.17
South West	2 532 019 (9)	4155 (13)	0.16
Scotland	2 629 517 (9)	2199 (7)	0.08
Wales	1 499 303 (5)	1419 (4)	0.09
Total	29 345 507	32 784*	0.11

*Where numbers do not sum to total, this is because of missing postcode or other information.

## How often have the participants been followed up?

The whole cohort has been contacted for a second time, Phase 2. All baseline responders were mailed between 1999 and 2002. Non-responders were mailed a postcard reminder and then followed up with a telephone call; 14 172 (40%) completed a follow-up health and lifestyle questionnaire similar to that used at baseline, and 12 453 (35%) also completed a 4-day food diary. There were some small differences in baseline characteristics by response status at Phase 2. For example, responders were slightly more likely to: be educated to degree level; have a lower body mass index (BMI); be less likely to smoke; eat less meat and more fruit and vegetables; and take dietary supplements, compared with non-responders (Table 2).

**Table 2.** Demographic, health and lifestyle characteristics of the UKWCS at baseline for the total sample; and split by those who responded or did not respond at Phase 2

Variable	Provided variable data at baseline, *n*	Baseline	Phase 2*	
			Responder	Non-responder
			(*n* = 14 172)	(*n* = 21 200)
Age (mean, SD)	34945	52.3 (9.4)	52.4 (9.1)	52.3 (9.5)
White ethnicity*, n* (%)	34372	33923 (98.7%)	13686 (98.6%)	20541 (98.5%)
Married or living as married, *n* (%)	34818	26115 (75%)	10653 (76.3%)	15462 (74.1%)
Highest educational qualification, *n* (%)				
Degree		8787 (27.2%)	3989 (30.6%)	4798 (24.9%)
A level		7949 (24.6%)	3386 (26.0%)	4563 (23.7%)
O level		10059 (31.1%)	3891 (29.8%)	6168 (32.0%)
No qualification		5525 (17.1%)	1779 (13.6%)	3746 (19.4%)
Paid job, *n* (%)	34308	20939 (61.0%)	8623 (62.4%)	12316 (60.1%)
Had any children, *n* (%)	31428	27053 (86.1%)	10775 (85.4%)	16278 (86.5%)
Physical exercise, h/day (mean/SD)	33444	0.3 (0.5)	0.3 (0.5)	0.3 (0.5)
BMI (mean, SD)	34013	24.5 (4.4)	24.2 (4.3)	24.7 (4.4)
Portions of fruit per day (mean, SD)	35365	5.3 (4.2)	5.5 (4.3)	5.1 (4.2)
Portions of vegetables per day (mean, SD)	35365	5.2 (3.0)	5.4 (3.0)	5.1 (3.0)
Food group: *n* (%)	32248			
Meat eaters		22808 (70.7%)	8821 (66.8%)	13987 (73.5%)
Fish eaters		4375 (13.6%)	1967 (14.9%)	2408 (12.7%)
Vegetarians		5065 (15.7%)	2423 (18.3%)	2642 (13.9%)
Daily smokers: *n* (%)	34319	2818 (8.2%)	825 (6.0%)	1993 (9.7%)
Alcohol consumption: *n* (%)	34568			
More than once a week		18032 (52.2%)	7221 (52.1%)	10811 (52.2%)
Never		3865 (11.2%)	1516 (10.9%)	2349 (11.4%)
Supplement taker *n* (%)	32117	18565 (57.8%)	7776 (60.2%)	10789 (56.2%)

*Percentages relate to variable numbers of questionnaire responses.

**Figure 2.**
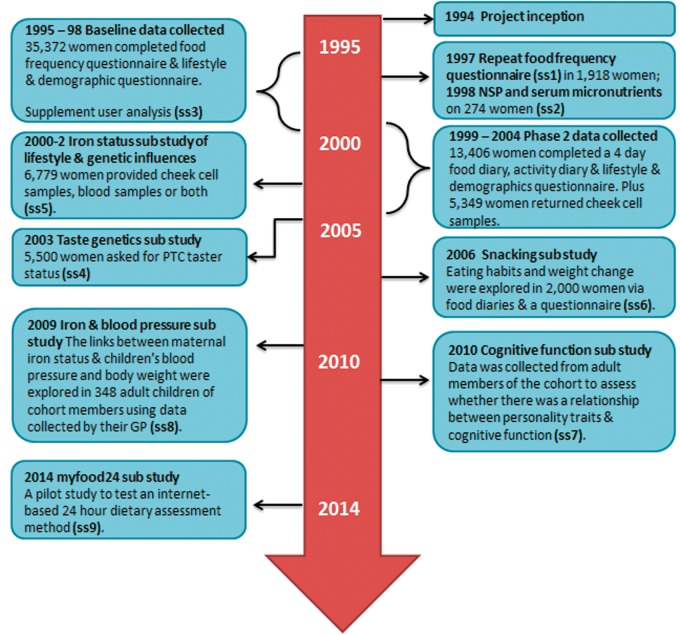
Timeline of UK Women’s Cohort Study data collection (ss, sub-study number).

Nine sub-studies have re-contacted samples of the cohort (Figure 2). Sub-studies 1 to 7 explore in-depth dietary measures or biomarkers of diet. Sub-studies 8 and 9 further expand the cohort into trans-generational measures.

### Dietary measures and biomarkers sub-studies

#### Repeat food frequency questionnaire

The first of these was a sub-group of 1918 women who completed a repeat FFQ, approximately 2 years after baseline. This allowed assessment of stability of dietary patterns, and an estimation of random measurement error in the FFQ.^9–11^

#### Non-starch polysaccharide intake and serum micronutrient concentrations

To investigate the effect of non-starch polysaccharide (nsp) on plasma nutrient concentrations, participants were restricted to those living within a 30-mile radius of Leeds, or a 1-h drive, to facilitate collection of blood samples. A total of 274 participants had blood samples analysed for a range of micronutrients. Higher levels of nsp were not associated with lower plasma concentrations of the micronutrients measured, even allowing for the higher nutrient levels generally found in high-fibre foods.^12^

#### Dietary supplement user sub-study

Supplement (*n* = 8409) and non-supplement users (*n* = 5413) were compared from the first half of the UKWCS baseline data. Supplement users had a healthier lifestyle profile and an adequate nutritional intake, suggesting that supplement users do not need to take supplements to meet a nutrient deficiency.^13–15^

#### Taste genetics sub-study

The Taste Genetics (TaGI) sub-study began in 2003. A sub-sample of 5 500 women who were ideal responders were mailed and asked to provide data regarding phenylthiocarbamide (PTC) taster status, food preference and food behaviour. PTC is a chemical that mimics the bitter taste sensation and is detectable in varying levels by different individuals. Respondents were categorized as ‘non-tasters’, ‘tasters’ or ‘supertasters’, based on their response to PTC-impregnated filter papers using a Labelled Magnitude Scale (LMS).^16^

#### Iron Status sub-study

The Iron Status sub-study was nested within Phase 2 data collection to determine the relationships between food and nutrient intakes, HFE genotype and iron status. Foods and nutrients associated with iron stores, with adjustment for gene mutations associated with haemochromatosis, were explored. A subgroup of 15 000 participants provided cheek cell samples by mail to assess two mutations linked to iron metabolism. All women who were homozygous or heterozygous for the C282Y gene mutation, along with a random sample of 3000 women, provided a blood sample for measurementof iron storage markers and DNA. Cheek cell samples, blood samples or both, were genotyped for C282Y andH63D mutations from 6779 women; 2489 women also had their iron status assessed using serum ferritin and transferrin saturation.^17^^,^^18^

#### Snacking sub-study^19^

A total of 3596 Phase 2 respondents with eating frequency coded from the first food diary in waves 1–4 (mailed between 24 April 1999 and 4 February 2000) were mailed a questionnaire and further 4-day food diary in 2006, to explore snacking habits and body weight status; 2253 women responded.

#### Cognitive function testing sub-study

The aims of this sub-study were to determine the acceptability of web-based cognitive function testing in a large cohort of older women, and to establish cross-sectional associations between traits and components of successful ageing. In 2010/11, a random sample of 2000 surviving women, who were not cancer registered, were invited to complete a questionnaire which included a personality assessment and an invitation to complete a reaction time task online. There were 920 questionnaires returned, a response rate of 46%; 426 (64%) additionally provided data on reaction time using an online task.

### Trans-generational research sub-studies:

#### Iron and Blood Pressure sub-study

In 2009, the Iron and Blood Pressure sub-study was conducted; sampling was linked to the Iron Status sub-study. The aim was to use Mendelian randomization to examine the relationship between maternal iron status [using maternal serum ferritin as the environmental measure of exposure (modifiable risk factor)] and maternal C282Y genotype as the instrumental variable, with offspring blood pressure and adiposity measures (body mass index and waist circumference) in adulthood. The non-exposed were randomly sampled from women with a wild-type genotype and one or more children. In all, 1686 children from C282Y carrier mothers and 1690 from wild-type mothers were contacted; 517 (17%) consented to take part, and 348 offspring of 277 mothers completed the study. Of these, 170 (49%) were children of C282Y mutation genotype mothers, of whom 12 were children of 11 homozygous mothers (aa), and 158 were children of heterozygous mothers (ag).^20^

#### myfood24 feasibility sub-study

In 2014–15, we are contacting a random sub-sample of participants who have completed Phase 2 data collection, and are still alive, and ask them to complete a new web-based 24-h dietary recall using the myfood24 tool.^21^^,^^22^ We also plan to explore intergenerational issues linked to dietary intake through recruitment of the women’s partners, their adult children and their adolescent grandchildren.

### Cohort consortia and other work including UKWCS

The UKWCS has also been included in a number of consortia. The United Kingdom Dietary Cohort Consortium has included the UKWCS in pooled analyses of dietary data collected using food diaries in seven prospective studies. This work has focused on diet in relation to risk of colorectal and breast cancer.^23–25^ The InterLace consortium of 14 studies, including the UKWCS, aims to identify markers of reproductive health and their inter-relationships over the life course among almost 120 000 women in seven countries.^26^ Publications from the UKWCS have contributed to the World Cancer Research Fund (WCRF) continuous updates project, contributing to reviews of breast cancer, as well as the Scientific Advisory Committee on Nutrition report on carbohydrates and cardiometabolic health.^27^^,^^28^

## What has been measured?

### Exposure measurements

The baseline questionnaire included a range of socio-economic, lifestyle and health status questions including self-reported anthropometric measures and weight change over time. Detailed reproductive history; information on past health experience, sibling and parental health as well as physical activity assessment were obtained. A 217-item food frequency questionnaire was included, based on the questionnaire used in EPIC,^29^ adapted for the large proportion of vegetarians included in this study. At Phase 2, the lifestyle and health questions were repeated. A 4-day food diary and 1-day activity diary were also collected, in addition to information on dietary supplement intake and cooking methods. Repeat self-reported waist and hip measures were taken (Table 3).

**Table 3.** Summary of primary variables collected in UKWCS; 697at baseline and 580 at Phase 2

Primary variable	Summary
Dietary intake	Food and nutrient intake in past 12 months (FFQ at baseline)
	4-d food diary (phase 2)
	Dietary patterns (self-defined and data-derived)
	Self-reported snacking frequency (ss6)
	Dietary supplement use (b, p2)
	Cooking habits (b, p2)
Anthropometric measures	Height, weight, waist, hip, blouse sizes, skirt sizes (self-reported) (b, p2)
	Weight: current, at 20, 30, 40, 50, 60, 70 years old, birthweight (b, p2)
	Loss or gain weight in past year (b)
Lifetime socioeconomic position	Self and partner socioeconomic status (based on job type/category) (b)
	Education (b)
	Alcohol consumption (b, p2)
Lifetime lifestyle	Smoking behaviour (b, p2)
	Physical activity (b, p2)
	Marital status (b)
	Hormone replacement therapy use (HRT) (b, p2)
	Contraceptive pill use (b, p2)
Health outcomes	Cancer and death reporting from HSCIC/NHSCR
	CHD, CVD, MI, stroke from HES & MINAP
	Self-reported illness (including specific cardiovascular-related disorders, intestinal disorders, cancers)
	Parental and sibling cancer/heart attack (p2)
	Menstrual and obstetric history (b, p2)
Biological samples collected	Cheek cell DNA samples (*n* = 5343) (ss4)
	Blood DNA samples (*n* = 2624) (ss4)
	Blood samples (*n* = 2485) (ss4)
	Serum samples (*n* = 2611) (ss4)
Cognitive function	Pilot data collection (*n* = 2000) (ss7):
	Internet-mediated reaction time task (Reimers & Stewart, 2007)
	2 personality inventories:
	• 48-item EPQ-R-S (Eysenck, Eysenck & Barrett, 1985)
	• 80-item ‘big 5’ adjective pairs (McCrae & Costa, 1985)

B, baseline; p2, Phase 2; ss, sub-study number; d, day; HSCIC, Health and Social Care Information Centre; NHSCR, National Health Service Central Register; HES, Hospital Episode Statistics; MINAP Myocardial Infarction National Audit Project; EPQ-R-S, short-form revised Eysenck personality questionnaire.

Phase 2 questions asked in detail about 17 different types of dietary supplements. More detailed supplement use was recorded in the 4-day diaries. Over 12 000 participants completed food diaries for 4 days, with a separate page for each day to record individual supplements taken including: brand; name; amount taken; and dosage. This information was captured into a database of supplements taken by participants, and matched via a drop-down menu against supplements listed in a supplement ingredient database. This enabled the allocation of ingredient amounts to the supplements taken. In total there were 3996 different marketed supplement types listed in the database and each was given a separate supplement identification code. This contained brand name, supplement descriptions, ingredient composition and units, which were obtained from product labels provided by participants, suppliers’ websites or provided directly from manufacturers upon request.

Although a relatively healthy cohort, around a quarter of the women were overweight and 10% obese. A third were not meeting the recommended intakes for fruit and vegetables and 80% had a low level of physical activity (Table 4).

**Table 4.** Risk factors for cancer and CVD in the UKWCS

Risk factor	Definition	Baseline (*n* = 35365)	Phase 2 (*n* = 14169)
Smoking status, *n* (%)	Current smoker	3810 (10.8%)	1183 (8.4%)
Obese, *n* (%)	BMI > 30 kg/m^2^	3353 (9.9%)	1442 (10.9%)
Overweight, *n* (%)	BMI > 25 kg/m^2^	8624 (25.4%)	3604 (27.3%)
Diabetes, *n* (%)	Self-report	646 (2.0%)*	183 (1.4%)
Hypertension, *n* (%)	Self-report	5763 (17.3%)	2168 (16.1%)
Cancer, *n* (%)	Self-report	2445 (7.5%)	928 (7.0%)
Heart attack, *n* (%)	Self-report	498 (1.5%)	161 (1.2%)
Angina, *n* (%)	Self-report	718 (2.2%)	233 (1.8%)
Stroke, *n* (%)	Self-report	264 (0.8%)	84 (0.6%)
Not meeting WCRF recommendations^46^ for cancer prevention:			
• Low physical activity	Active <30 min/day	28332 (80%)	11514 (81%)
• High energy intake	125 kcal/100 g or more	8067 (23%)	3257 (23%)
• Sugar-sweetened beverage (SSB) consumer	SSBs once a week or more	12003 (35%)	4505 (32%)
• Low fruit and vegetable intake	< 400 g/day vegetables & fruit	10772 (31%)	3846 (27%)
• High alcohol	> 1 unit per day	11774 (32%)	4545 (32%)

### Outcome measurement

Deaths and cancer registrations for the cohort are being recorded. Cause of death was coded in the International Classification of Diseases (ICD) 9 and now in 10. Prevalent cancers were identified from knowledge of pre-existing cancers from the NHSCR/HSCIC data linked to the baseline questionnaire date. Any cases occurring after the date of the questionnaire return are counted as incident. Cancer diagnoses are registered under ICD codes by local cancer registries and collated by the National Health Service Central Register, now the HSCIC. Cancer and death registrations for women in UKWCS are extracted quarterly by the NHSCR/HSCIC. This information is linked to UKWCS identification codes. Cardiovascular mortality records for participants have also been identified from the national registry data; 258 fatal cardiovascular disease (CVD) cases [130 stroke, 128 coronary heart disease (CHD)] were observed over an average follow-up period of 14.3 years.^30^ In addition, Hospital Episode Statistics (HES) and the Myocardial Infarction National Audit Project (MINAP) have also been linked to the UKWCS to provide information on CHD and stroke cases^31^ (Table 5).

**Table 5.** Cancer incidence and CVD outcomes in the UKWCS (follow up from 1998)

Cancer incidence: until Dec 2013	Cases/35372	Percent
Breast cancer	1579	4.46
Ovarian cancer	241	0.68
Uterine cancer	231	0.65
Cervical cancer	34	0.1
Bladder cancer	79	0.22
Colorectal cancer	491	1.39
Oesophageal cancer	66	0.19
Stomach cancer	38	0.11
Pancreas cancer	102	0.29
Lung cancer	248	0.7
Kidney cancer	71	0.2
CVD incidence: until June 2011*		
Total CVD	1162	3.29
Total CHD	812	2.30
All acute coronary syndromes (myocardial infarction)	392 (236)	1.11 (0.67)
Chronic heart diseases	573	1.62
Total stroke	388	1.10

*Cancer data are provided by HSCIC and CVD data by HES and MINAP sources.

### Stored samples

DNA is available from stored cheek cell samples on 5434 women and from blood samples for 2624 women. Blood samples are available from 2485 and plasma samples from 2576 women. Samples are from 2005 and stored at −20°C.

## What has it found? Key findings and publications

### Diet and breast cancer outcomes

Findings have used baseline FFQ data to provide evidence for the relationship between risk of breast cancer and dietary intakes. The study has demonstrated a positive association between meat intake and risk of breast cancer.^32^ Women, both pre- and postmenopausal, who consumed the most meat had the highest risk of breast cancer. The hazard ratio per extra 50 g/day was 1.11 [95% confidence interval (CI) 1.04 to 1.18] for total meat (*P*_trend_ = 0.001), and for processed meat 1.59 (95% CI 1.22 to 2.06; *P*_trend_ < 0.001). Larger effect sizes were found in postmenopausal women for all meat types compared with premenopausal women. An inverse association for risk of breast cancer with total fibre and cereal fibre intake has been demonstrated in premenopausal, but not postmenopausal women.^9^ The top quintile of fibre intake was associated with a hazard ratio of 0.48 (95% CI 0.24 to 0.96) compared with the lowest quintile. Premenopausally, fibre from cereals was also inversely associated with risk of breast cancer (P_trend_ = 0.05).

Exploration of common dietary patterns found no important associations with risk of premenopausal breast cancer, although a fish-eating dietary pattern that excludes meat from the diet may confer some benefit with regard to risk of postmenopausal breast cancer.^33^ In addition, no strong association between the risk of breast cancer and the consumption of either a Mediterranean-type diet or one characterised by adherence to the WHO Healthy Diet Index was observed.^34^

An exploration of dietary acrylamide intake in relation to breast cancer risk in the cohort found no evidence of association. A weak association may exist with premenopausal breast cancer, but this requires further investigation.^35^ Spatial analysis utilising postcode information has shown variation in breast cancer incidence and variation by dietary pattern. In postmenopausal women, a positive association exists between weight status and risk of breast cancer incidence.^36^^,^^37^

Other work is now being undertaken exploring diet in relation to colorectal and female reproductive hormone cancers. A full list of publications associated with the UKWCS can be found on the study website www.ukwcs.leeds.ac.uk.

### Diet and cardiovascular disease outcomes

Analysis of fatal CHD, stroke or CVD risk in the full sample found no association with total dietary fibre and fibre from different food sources.^30^ However, there was a possible protective association for cereal sources of fibre on fatal stroke risk in overweight women, HR 0.80 (95 % CI 0.65 to 0.93); *P* < 0.01. Total fibre intake was associated with total (fatal plus non-fatal) stroke events and the HR per 6 g/day total fibre intake was 0.89 (95% CI 0.81 to 0.99).^31^

### Other health and lifestyle outcomes related to diet

Other results have focused on particular nutrients from the diet or supplements linked to non-cancer outcomes. Haem, but not non-haem or total iron, is associated with serum ferritin concentrations.^17^ Postmenopausally, we found a strong interaction between genotype and haem iron intake on iron status. Postmenopausal women eating a diet rich in haem iron and who were C282Y homozygotes had the highest serum ferritin concentrations. Women with only one copy of the C282Y mutation and H63D homozygotes and heterozygotes have similar serum ferritin concentrations to wild type.^18^ We also found no association between maternal iron status and the participant’s adult offspring's blood pressure and adiposity using both multivariable regression and instrumental variable modelling.^20^ High non-starch polysaccharide intakes are also not associated with poorer micronutrient status within the broad range of intakes observed in this cohort.^12^ Our findings mean that current guidelines on healthy eating remain valid.

Women with personal or family histories of some cardiovascular or intestinal disorders were more likely to take supplements containing vitamin C, though not necessarily at high doses. High-dose vitamin C intake was linked to healthier behaviours and a history of breast cancer, total cancer and other illnesses within close family members.^38^ Supplement users believed more strongly than non-users that taking dietary supplements would stop them getting ill and help them to be healthy.^13^ However, we found that women taking supplements did not experience a lower risk of breast, colorectal or total cancer.^39^

Diets of the women have been matched to food-cost databases; results show that a healthier diet, defined by a healthy diet index^40^ and by data-driven dietary patterns scored against the Eatwell Plate,^41^ is more expensive. Diet cost and dietary diversity were positively linked to healthiness of the diet. The healthiest dietary pattern was double the price of the least healthy, £6.63/day and £3.29/day, respectively (at 2004 prices). Those with higher education and a professional or managerial occupation were more likely to consume a healthier diet.^41^

## What are the main strengths and weaknesses?

One of the main strengths of the study is the recruitment of participants into the cohort with a wide range of exposure to different dietary patterns; this decreases measurement error bias and increases power.^42–44^ In addition, diet has been assessed by both FFQ and 4-day food diary. Extensive lifestyle information has also been collected. The focus on women has allowed adequate power for exploration of diet and chronic disease outcomes in this particular group. However, the cohort does not represent a random sample of middle-aged British women since the cohort was healthier than the general population.^7^ Nevertheless, the over-sampling of vegetarians does not make results unrepresentative because we have re-weighted results to the proportion of vegetarians in the UK population, allowing wider generalization of findings. Attrition between baseline and Phase 2 was high. There are larger numbers in all groups than alternative dietary data in the UK such as the National Diet and Nutrition Survey. The FFQ method is an approach to measure diet and commonly used in large cohort studies; it is limited by a range of potential methodological biases. These include the foods listed, frequency of consumption and limitations concerning the assessment of portion size.^45^ In-depth food diary data are also available, and this type of detailed dietary information is rarely available for large cohort studies. It is possible to undertake sub-studies on samples of the cohort.

## Can I get hold of the data? Where can I find out more?

All collected source data are maintained and stored by the Nutritional Epidemiology Group, University of Leeds. Specific proposals for collaboration are welcomed. Further information can be found on the UKWCS website [www.ukwcs.leeds.ac.uk] or via email to [ukwcs@leeds.ac.uk] or [j.e.cade@leeds.ac.uk].

UK Women’s Cohort Study (UKWCS) profile in a nutshell

- UKWCS is a large cohort of British women exploring diet and lifestyle in relation to chronic disease outcomes.
- A total of 35 372 women aged 35–69 years were recruited at baseline, 1995–1998, from England, Scotland and Wales.
- The whole cohort has been contacted for a second time, between 1999 to 2002: 14 172 (40%) completed a follow-up questionnaire, and 12 453 (35%) also completed a 4-day food diary. In addition, nine sub-studies have re-contacted samples of the cohort. All women are registered with HSCIC for cancer and death notifications.
- The dataset comprises a wide range of variables (697 from baseline; 580 from Phase 2). These include: dietary intake; anthropometric measures; socioeconomic status; lifetime lifestyle; and health outcomes. Biological samples including blood and DNA and cognitive function measures are available on a sub-set.
- Specific proposals for collaboration are welcomed. Further information can be found on the UKWCS website [www.ukwcs.leeds.ac.uk] or via email to [ukwcs@leeds.ac.uk] or [j.e.cade@leeds.ac.uk].

## Funding

The UKWCS was originally funded by the World Cancer Research Fund. Other support has been received from the Food Standards Agency (grant NO5 023), the Medical Research Council (ref: G1100235/1), the Wellcome Trust (Fellowship WT87789), the Economic and Social Research Council, Kelloggs Sales and Marketing UK Ltd. The open access fee was funded by University of Leeds.
